# Single nucleotide polymorphism barcoding of cytochrome c oxidase I sequences for discriminating 17 species of Columbidae by decision tree algorithm

**DOI:** 10.1002/ece3.3045

**Published:** 2017-05-23

**Authors:** Cheng‐Hong Yang, Kuo‐Chuan Wu, Hans‐Uwe Dahms, Li‐Yeh Chuang, Hsueh‐Wei Chang

**Affiliations:** ^1^ Department of Electronic EngineeringNational Kaohsiung University of Applied SciencesKaohsiungTaiwan; ^2^ Graduate Institute of Clinical MedicineKaohsiung Medical UniversityKaohsiungTaiwan; ^3^ Department of Computer Science and Information EngineeringNational Kaohsiung University of Applied SciencesKaohsiungTaiwan; ^4^ Department of Biomedical Science and Environmental BiologyKaohsiung Medical UniversityKaohsiungTaiwan; ^5^ Department of Chemical Engineering and Institute of Biotechnology and Chemical EngineeringI‐Shou UniversityKaohsiungTaiwan; ^6^ Institute of Medical Science and TechnologyNational Sun Yat‐sen UniversityKaohsiungTaiwan; ^7^ Department of Medical ResearchKaohsiung Medical University HospitalKaohsiungTaiwan; ^8^ Research Center for Natural Products and Drug DevelopmentKaohsiung Medical UniversityKaohsiungTaiwan

**Keywords:** COI, Decision tree, SNP barcode, species discrimination, species tag

## Abstract

DNA barcodes are widely used in taxonomy, systematics, species identification, food safety, and forensic science. Most of the conventional DNA barcode sequences contain the whole information of a given barcoding gene. Most of the sequence information does not vary and is uninformative for a given group of taxa within a monophylum. We suggest here a method that reduces the amount of noninformative nucleotides in a given barcoding sequence of a major taxon, like the prokaryotes, or eukaryotic animals, plants, or fungi. The actual differences in genetic sequences, called single nucleotide polymorphism (SNP) genotyping, provide a tool for developing a rapid, reliable, and high‐throughput assay for the discrimination between known species. Here, we investigated SNPs as robust markers of genetic variation for identifying different pigeon species based on available cytochrome c oxidase I (COI) data. We propose here a decision tree‐based SNP barcoding (DTSB) algorithm where SNP patterns are selected from the DNA barcoding sequence of several evolutionarily related species in order to identify a single species with pigeons as an example. This approach can make use of any established barcoding system. We here firstly used as an example the mitochondrial gene COI information of 17 pigeon species (Columbidae, Aves) using DTSB after sequence trimming and alignment. SNPs were chosen which followed the rule of decision tree and species‐specific SNP barcodes. The shortest barcode of about 11 bp was then generated for discriminating 17 pigeon species using the DTSB method. This method provides a sequence alignment and tree decision approach to parsimoniously assign a unique and shortest SNP barcode for any known species of a chosen monophyletic taxon where a barcoding sequence is available.

## 1 INTRODUCTION

The original idea of DNA barcoding was to use a short DNA sequence as a species‐specific marker for species identification and authentication (Hebert, Cywinska, Ball, & deWaard, 2003). It differs from molecular phylogeny approaches as the main purpose is not to analyze evolutionary relationships but to identify an unknown species within a known phylogenetic classification system (a monophylum) using DNA sequences (Kress, Wurdack, Zimmer, Weigt, & Janzen, 2005).

The DNA barcoding technique utilizes a short DNA sequence of the genome that provides enough variation at the species level to unequivocally define a taxon at the species level (http://www.barcodeoflife.org/content/about/what-dna-barcoding). A suitable barcoding gene is commonly of unique reproducibility, sequence versatility, and comparability among different species belonging to the same major kingdom, like animals, plants, or fungi (Yan et al., 2013). For animals and some other eukaryotes, the most successful results were provided by the mitochondrial gene cytochrome c oxidase I (COI), which is the standard gene region in the range of 650 base pairs (bp) (Hebert et al., 2003). Within vertebrate animals, COI was proposed as a potential barcode for the identification of 260 North American bird species (Hebert, Stoeckle, Zemlak, & Francis, 2004). Such information was applied in food authentication and safety (Vandamme et al., 2016) as well as for forensic purposes (Bell, Burgess, Okamoto, Aranda, & Brosi, 2016; Desmyter & Gosselin, 2009; Dubey, Meganathan, & Haque, 2011). Although the COI sequence is conventionally used as an unarbitrary barcode for the discrimination between eukaryotic and animal species, its major shortcoming is that it takes substantial memory and processing time for computational comparisons, particularly when dealing with large data. Such large data are increasingly available with metagenomic approaches to species diversity, even if only using a single promising barcoding gene, like COI (Gao, Jia, & Kong, 2016). Therefore, reducing the amount of noninformative data for computational analysis in species identification remains a challenge with promising applications.

Several genetic markers were developed in the past for the purpose of species and population characterization (Grover & Sharma, 2016). Restriction fragment length polymorphisms (RFLPs) were among the first genomic markers. RFLPs have the disadvantage of being complex, costly, and showing a comparatively low rate of polymorphism. Often considered as a second generation of genomic markers, SSRs (simple sequence repeats) are easy to obtain at lower cost showing a higher polymorphism rate (Gao et al., 2016). Single nucleotide polymorphisms (SNPs) are considered as the third‐generation of markers. With the development of next‐generation sequencing (NGS) technology and low‐cost genome sequencing, a large number of SNPs have recently been identified with the microarray technology based on a standardized protocol (Unterseer et al., 2014). SNP arrays comprise loci with unique positions along chromosomes or genomes, thereby largely avoiding the confusion associated with multiple sequence variants but still at comparatively high costs, for example, specific patterns of SNP. A bioinformatics‐based approach in reducing the computational database to an amount of informative gene sequences would be helpful to deal with this problem. Such an approach would effectively reduce the complexity of a given barcoding sequence information in terms of SNP. An economic and easy to apply molecular barcode with a high‐throughput possibility is required for determining species for above applications. Here, we show the development of a minimal set of SNP markers that is robust enough to fingerprint a diverse collection of species. In our previously developed software Seq‐SNPing (Chang et al., 2009), the SNPs were easy to identify after sequence alignment.

In this article, we propose a decision tree‐based SNP barcoding (DTSB) algorithm that automatically generates barcodes for species identification through a decision tree approach. This will facilitate to discriminate biota at species level based on a machine learning technique to analyze given COI sequences from 17 pigeon species. We hypothesize that SNPs from aligned COI sequences of different know species can be used as a new of straightforward way to strip barcoding sequence information from nonvariable and noninformative information to gain shortest variable bp information allowing speedy computational comparisons for the purpose of species discrimination.

## 2 MATERIAL AND METHODS

### 2.1 Data sources

Seventeen COI sequences of the bird family Columbidae containing four genera were used in this study. These data were obtained from GenBank; the details are provided in Table 1.

**Table 1:** 17 COI sequences of bird species belonging to the pigeon family (Columbidae) from GenBank

Family	Genera	Species name	Length (bp)	Accession no.
Columbidae	*Columbina*	*C. talpacoti*	694	FJ027432.1
		*C. picui*	694	FJ027428.1
		*C. passerina*	681	DQ433537.1
		*C. inca*	674	DQ433529.1
	*Columba*	*C. oenas*	722	GU571344.1
		*C. rupestris*	694	GQ481615.1
		*C. palumbus*	694	GQ481607.1
		*C. livia*	694	GQ481606.1
	*Zenaida*	*Z. auriculata*	694	FJ028598.1
		*Z. macroura*	697	DQ434834.1
		*Z. asiatica*	652	DQ433271.1
	*Patagioenas*	*P. picazuro*	694	FJ027979.1
		*P. maculosa*	694	FJ027973.1
		*P. cayennensis*	694	FJ027970.1
		*P. araucana*	694	FJ027968.1
		*P. flavirostris*	682	DQ433887.1
		*P. fasciata*	680	DQ433886.1

### 2.2 Decision trees

**Figure 1:**
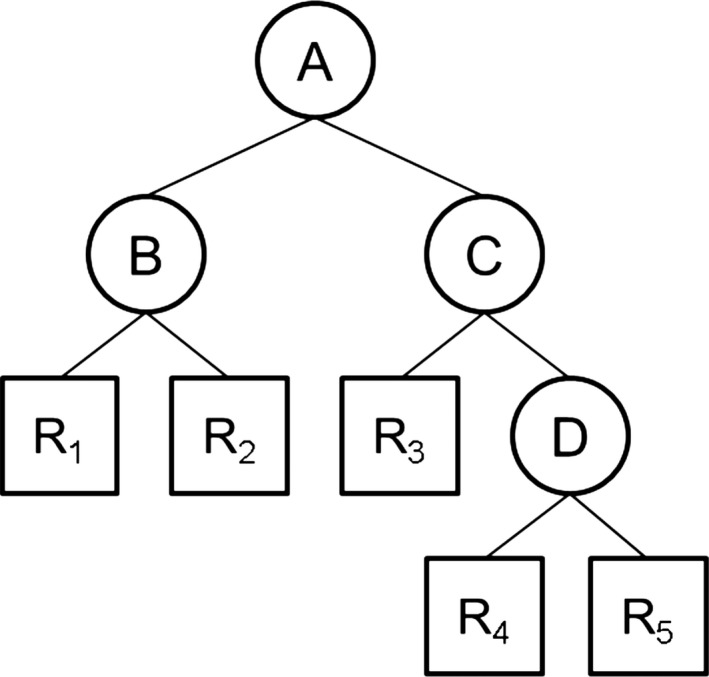
An illustration of a simple decision tree. The circle represents decision nodes (parent nodes), which is a decision‐maker to make a choice according to decision rules. The rectangle represents leaf nodes (child nodes)

An introduction of tree‐like structures (graph or model) of decision tree algorithm is shown in Figure 1. This simplified algorithm recursively implements from top (root, see circle A of Figure 1) to bottom (leaf nodes, see rectangle R_1_ to R_5_ of Figure 1). The procedure of generating trees begins with the root, each node in the tree is according to the rule, and it is determined which path from the decision node to another decision node or left node is taken. This procedure continues until arriving at a left node. Two common measures are used: Entropy and Gini index. In general, the decision tree criterion is used for the decision rule that is splitting the decision node to branch into a leaf that is called information gain measurement, which is expressed as (formula (1)):

(1) Gain(A,S)=Info(S)−E(S)

where Gain(*A*,* S*) is an estimate of the amount of set of uncertainty (*S*) and set of attributes (*A*). The entropy *E*(*S*) is defined as follows (formulas (2) and (3)):

(2) $$E\left(S\right)=\sum_{i=1}^{k}\left(\frac{\left(p_{i}+n_{i}\right)}{\left(p+n\right)}\right)\cdot \text{Info}\left(p_{i},\;n_{i}\right)$$

where *k* is the number of attribute, *S *= *p *+ *n*,* p* is the number of samples with positive target, and *n* is the number of samples with negative target.

(3) $$\text{Info}\left(p,\;n\right)=-D_{p}\log_{2}\left(D_{p}\right)-D_{n}\log{}_{2}\left(D_{n}^{}\right)$$

where, $D_{p}=\frac{p}{\left(p+n\right)},\;\;D_{n}=\frac{n}{\left(p+n\right)}$ $D_{n}=\frac{n}{\left(p+n\right)}$

The Gini index Gini(*e*) is defined as (formula (4)):

(4) $$\text{Gini}\left(e\right)=1-\sum_{i}P{\left(i|e\right)}^{2}$$

where *P*(*i*|*e*) is conditional probability of category *i* at node *e* of the tree and defined as follows (formulas (5), (6), and (7)):

(5) $$P\left(i|e\right)=\frac{P(i,e)}{P(e)}$$

(6) $$P\left(i,e\right)=\frac{P(\pi_{i}\cdot N_{i}\left(e\right))}{N_{i}}$$

(7) $$P\left(e\right)=\sum_{i}P(i,e)$$

where π_*i*_ is the prior probability value for class *i*,* N*_*i*_(*e*) is the number of records in class *i* of node *e*, and *N*_*i*_ is the number of records of class *i* in the root.

In Gini index, when the value of Gini(*e*) is bigger, the distribution of class of samples is average. Otherwise the distribution of class of samples is unaverage. In a decision tree, three main parameters in the decision tree include (1) the tree constraints, (2) the splitting criterion, and 3) the tree pruning method (De Mántaras, 1991; Gelly, Chiche, & Gracy, 2005; Quinlan, 1986).

### 2.3 TSB approach

**Figure 2:**
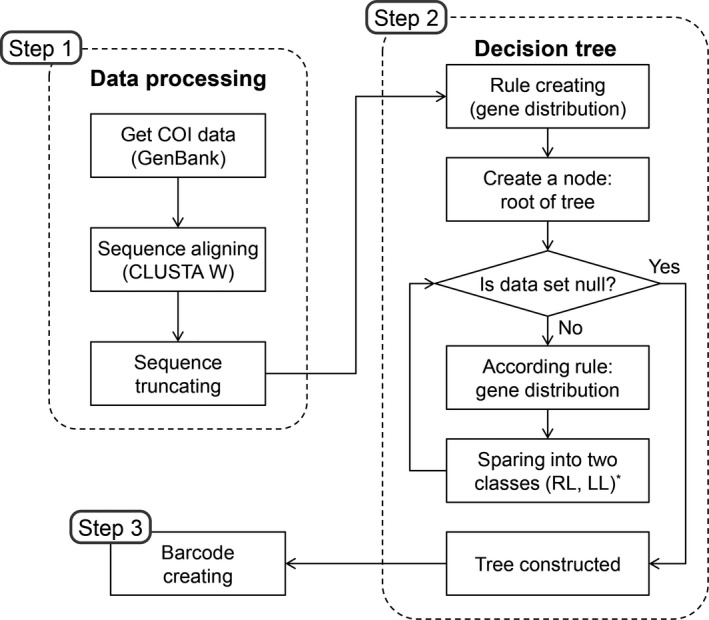
The flowchart of the DTSB approach. *RL: Right leaf node, LL: Left leaf node

In this study, we propose a novel approach to implement the species identification process, called decision tree‐based SNP barcoding (DTSB) method. The DTSB method is generating a shorter barcode through decision tree construction using COI DNA sequences. The flowchart of the DTSB approach is given as follows (Figure 2): Step 1) data processing, Step 2) decision tree making, and Step 3) barcode sequence creation.

#### 2.3.1 TSB step 1) data processing

Several COI data were collected from GenBank. Because the variation of the length of COI in different accession numbers, they were aligned with ClustalW program (Tamura et al., 2011), and the resulting sequences were truncated to keep the same sequence length for further processing.

#### 2.3.2 TSB step 2) decision tree making for barcoding

Support the data ***X*** are composed of the alignment with *N* sequences (from each species) of the same length of *M* nucleotides, and it can be written as follows (formula (8)):

(8) $$X=\left[\begin{array}{ccccc} x_{1,1} & x_{1,2} & x_{1,3} & \cdots & x_{1,M}\\ x_{2,1} & x_{2,2} & x_{2,3} & \cdots & x_{2,M}\\ \vdots & \vdots & \vdots & \ddots & \vdots \\ x_{N,1} & x_{N,2} & x_{N,3} & \cdots & x_{N,M} \end{array}\right]$$

When the nucleotides A, C, G, and T are used in the matrix ***X***, we obtain the distribution ***D*** of nucleotides in each position *p *∈ [1, *M*] of ***X***, and the distribution ***D*** is represented by (formulas (9) and (10)):

(9) $$D=\left[\begin{array}{ccc} f_{A1} & f_{A2} & f_{A3}\cdots f_{AM}\\ f_{C1} & f_{C2} & f_{C3}\cdots f_{CM}\\ f_{G1} & f_{G2} & f_{G3}\cdots f_{GM}\\ f_{T1} & f_{T2} & f_{T3}\cdots f_{TM} \end{array}\right]$$

where

(10) $$f_{ip,\;\;i\in \left\{A,\;C,G,\;T\right\}}=\sum_{k}^{N}\left(x_{k,p}|i\right)$$

In the decision tree, the rules are designed to distinguish species and subgroups into two sides (right and left leaves) based on the score ***S*** in each position of sequences. The ***S*** is represented as follows (formula (11)):

(11) $$S=\left[\begin{array}{cc} \begin{array}{ccc} {\text{score}}_{1} & {\text{score}}_{2} & {\text{score}}_{3} \end{array} & \begin{array}{cc} \cdots & {\text{score}}_{M} \end{array} \end{array}\right]$$

where the score at the position (*p*), namely ${\text{score}}_{p}$, is calculated as (formulas 12‐15):

(12) $${\text{score}}_{p}=\frac{{\text{mid}}_{p}-\text{di}\;f\;f_{p}}{{\text{mid}}_{p}}+{\text{weight}}_{p}$$

where

(13) $${\text{mid}}_{p}=\frac{\text{number of data set in node}}{2}$$

and

(14) $${\text{diff}}_{p}=\;\min_{\;\;i\in \left\{A,\;C,G,\;T\right\}}\left\{\left|{\text{mid}}_{p}-f_{ip}\right|\right\}$$

and

(15) $${\text{weight}}_{p}=\left\{\begin{array}{cc} 0, & \text{if the number of appeared nucleotide type is 1}\\ 1, & \text{if the number of appeared nucleotide type is 2}\\ 0.66, & \text{if the number of appeared nucleotide type is 3}\\ 0.33, & \text{if the number of appeared nucleotide type is 4} \end{array}\right.$$

Consequently, the species can be divided into two sides according to the score calculation for each score_*p*_. The rest nodes at different levels are performed in the same manner, and the tree is finally constructed.

**Figure 3:**
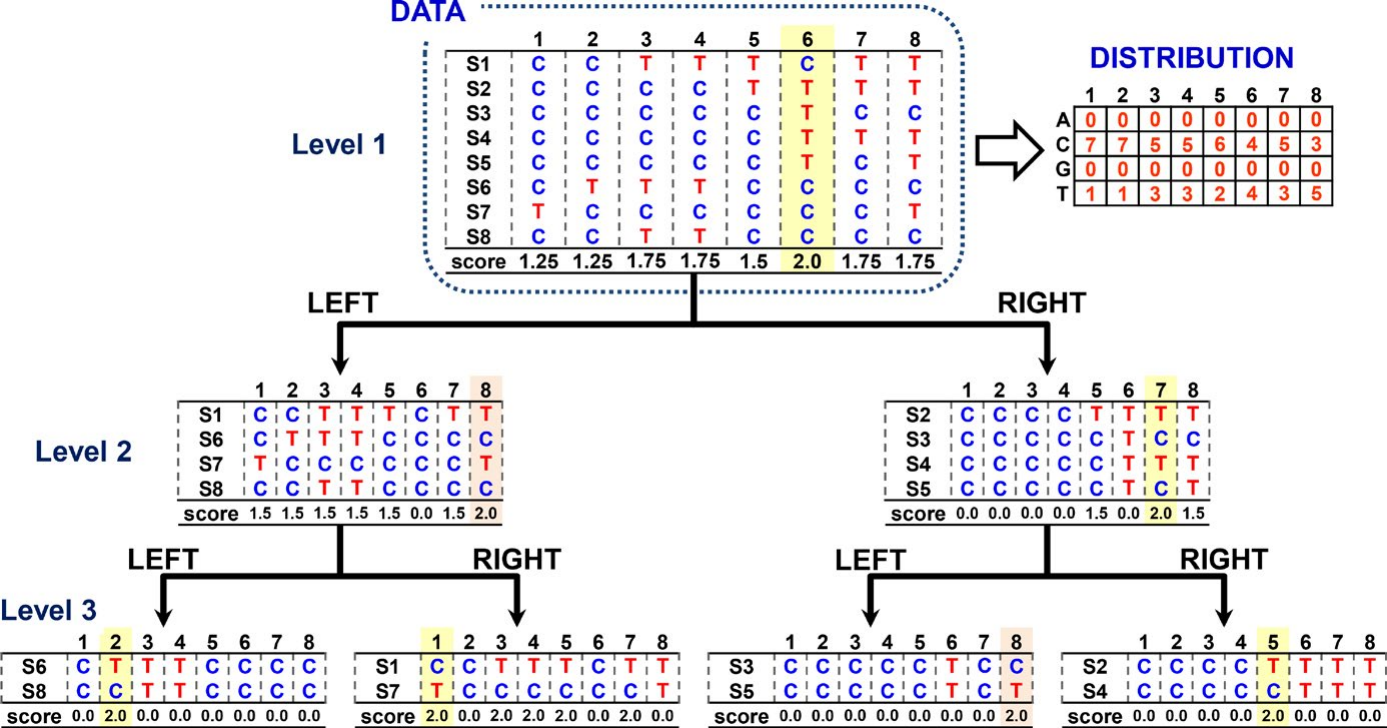
Example of decision tree making in DTSB. On each level, decision trees are made, and left and right sides are generated. S1–S8 indicate the eight sequences with the lengths of eight nucleotides collected from eight species. The number at the top of each table indicates the order of nucleotides. The number at the bottom of each table indicates the scores for each position, for example, score_*p*_

Assuming that we get the “data” of 8 sequences (species) of a length for eight nucleotides (Figure 3), then the “distribution” is counted from “data” and the scores S (score_*p*_) are calculated using formula 12. For example, the positions *p*_1_ and *p*_6_ in level one has eight sequences (species); therefore, the mid_1_ and mid_6_ are $\frac{8}{2}=4$ (formula 13) and the diff_1_ and diff_6_ are calculated as follows (formula 14):

$${\text{diff}}_{1}=\min\left\{\begin{array}{c} \begin{array}{c} f_{A1}=\left|4-0\right|=4\\ f_{C1}=\;\left|4-7\right|=3 \end{array}\\ \begin{array}{c} f_{G1}=\;\left|4-0\right|=4\\ f_{T1}=\;\left|4-1\right|=3 \end{array} \end{array}\right.=3$$

and

$${\text{diff}}_{6}=\min\left\{\begin{array}{c} \begin{array}{c} f_{A6}=\left|4-0\right|=4\\ f_{C6}=\;\left|4-4\right|=0 \end{array}\\ \begin{array}{c} f_{G6}=\;\left|4-0\right|=4\\ f_{T6}=\;\left|4-4\right|=0 \end{array} \end{array}\right.=0$$

where there are two types in *p*_1_ and *p*_6_ (C and T); hence, weight 1 and weight 6 are 2 (formula (14)). The scores are calculated as follows (formula 12):

$${\text{score}}_{1}=\;\frac{4-3}{4}+1=1.25$$

and

$${\text{score}}_{6}=\;\frac{4-0}{4}+1=2$$

Therefore, we can get all scores of positions *p*_1_ ~ *p*_8_ (see Figure 3) and the maximum score in position *p*_6_ is obtained at level one. All sequences are subgrouped into “left” and “right” sides as branches according to nucleotides (here it is C and T). Subsequently, the subtrees follow the same procedure as described above until the end of tree. This way, the positions *p*_1_, *p*_2_, *p*_5_, *p*_6_, *p*_7_, and *p*_8_ are found (Figure 3). The position *p*_8_ is chosen twice, that is, (1) the left side of level 2 and (2) the right side of level 2 and its left side of level 3. Therefore, short barcode sequences are sometimes available using DTSB.

#### 2.3.3 TSB step 3) generating barcode sequences

The code 128 (standard) of one dimension barcode is commonly used for alpha‐numerical or numerical applications only. We use the website tool (http://www.barcode-generator.org/) to create barcode images.

## 3 RESULTS

**Figure 4:**
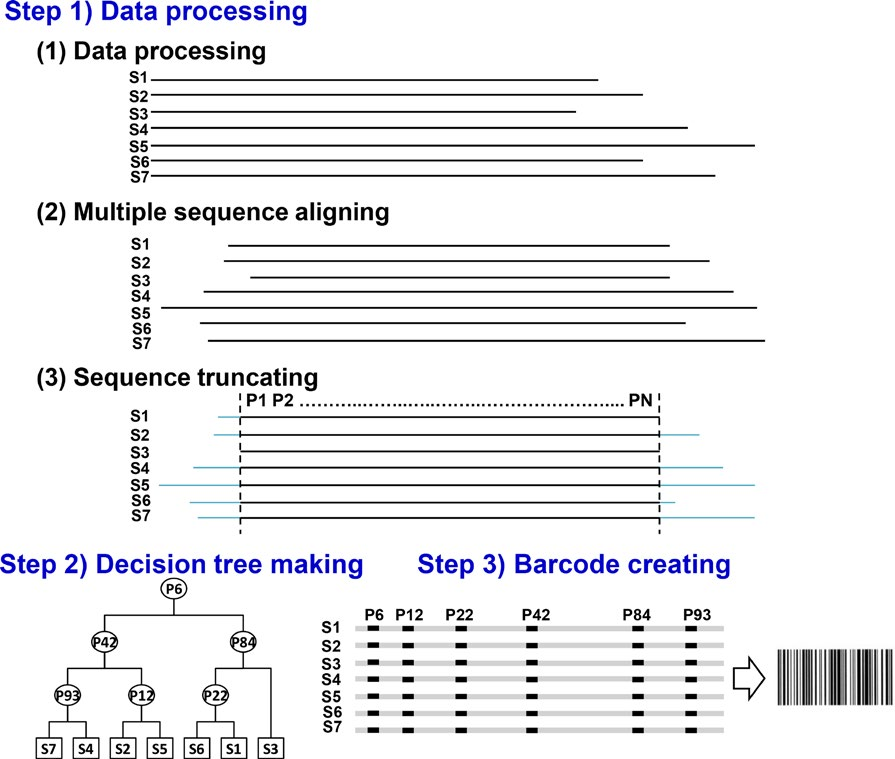
Illustration of DTSB approach. Three steps are processed to perform the DTSB method. S1–S7 indicate the example sequences from different species. In step 1, data processing is performed. Light blue lines in step 1 (3) indicate the protruding sequence after alignment. P1–PN indicate the position numbers for the sequence of common COI regions (Fig. S3) after trimming. In step 2, the decision tree making is performed. Subsequently, the species‐specific grouping is generated. The P numbers indicate the position numbers, and the S number indicates the example sequences. In step 3, barcode creating is performed. The SNPs identified from the decision tree are visualized and used to generate species‐specific SNP barcoding patterns

For the DTSB approach, the results of each step are obtained as follows (Figure 4).

### 3.1 Step 1) data processing

Step 1.1. COI data collection: Seventeen COI sequences of bird family Columbidae were collected and aligned as shown in Fig. S1 (Supporting information).
Step 1.2. *Aligning multiple sequences:* After performing MEGA 6 (Tamura, Stecher, Peterson, Filipski, & Kumar, 2013), the alignment of these seventeen COI sequences is shown in Fig. S2 (Supporting information).
Step 1.3. *Sequence truncation:* Because the length of COI for different species commonly differs, the sequence of the 5′ and 3′ ends of some COI sequences may be protruding and needs further trimming to generate equal bp lengths with blunt ends. Finally, a common region of the COI sequences was identified as shown in Fig. S3 (Supporting information). This common sequence was then used as the reference sequence for nucleotide position numbering.

### 3.2 Step 2) decision tree making

**Figure 5:**
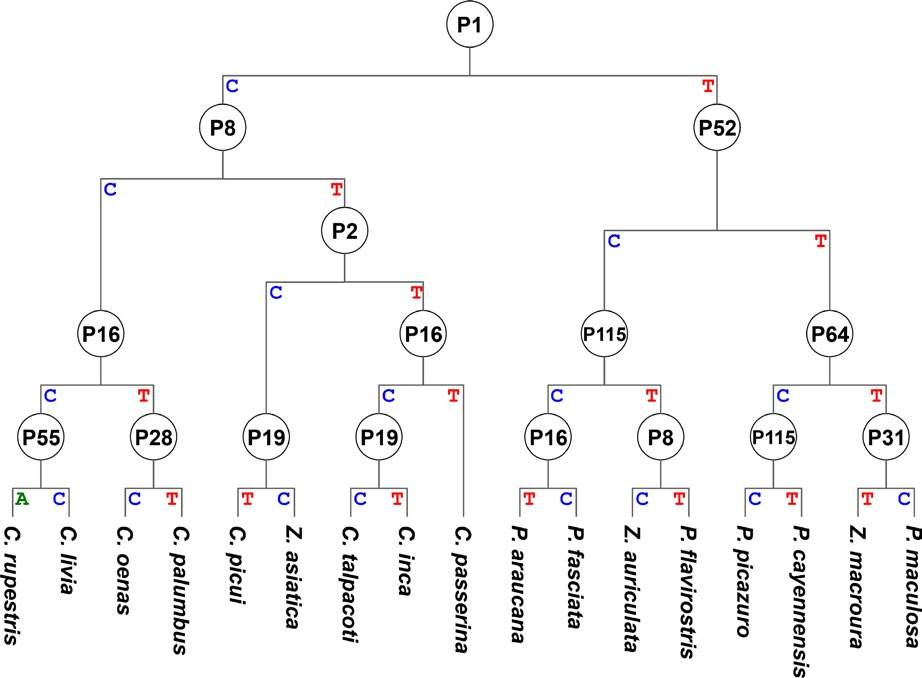
Decision tree outcome of the DTSB approach. P1–PN in each circle indicate the position numbers for the sequence of common COI regions (Fig. S3) after trimming. The letter in each turn indicates the nucleotide for grouping. Collection of the different levels becomes the SNP barcode for each test species as shown in Figure 6a

The decision rule was used to construct a decision tree according to the COI gene distribution sparing into two classes as shown in Figure 5. Based on the alignment‐generated SNP pattern, the tested 17 species were discriminated from each other using our proposed decision tree algorithm.

### 3.3 Step 3) generating barcode sequences

**Figure 6:**
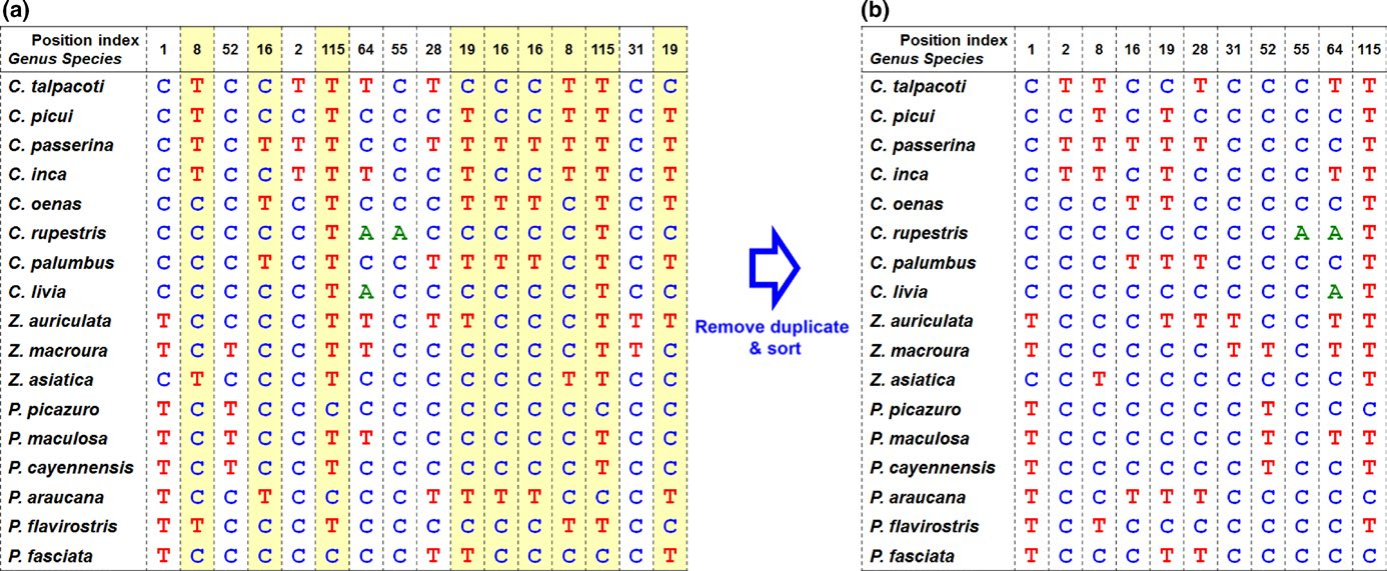
SNP barcode creation using the DTSB approach. (a) The SNP barcode from the decision tree outcome of the DTSB approach. The color background indicates that the nucleotide is repeatedly chosen. (b) The sorted SNP barcode. The position numbers were sorted from small to high, and the corresponding nucleotides were moved together. Repeated nucleotides are processed to keep only one nucleotide for the same position. The sorted SNP barcode is transformed into barcoding patterns as shown in Figure 7

**Figure 7:**
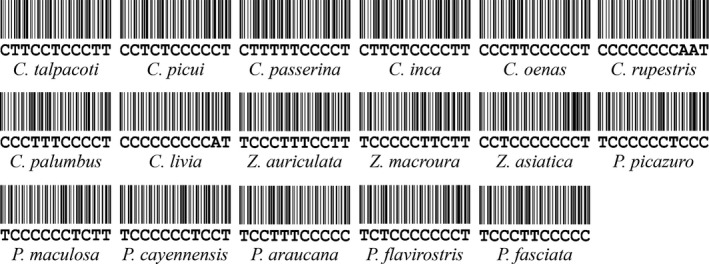
SNP barcodes for 17 Columbidae species

Finally, species‐specific COI SNPs generated from the decision tree algorithm were visualized into SNP barcode as shown in Figure 6a. However, the SNP pattern generated from decision tree was listed in the order of decision levels. The selected SNP from the top level was designed to appear first. The level next to the top level appeared next and so on. Accordingly, the number order of selected SNP appeared randomly. After sorting, species‐specific SNP barcodes were listed in the order of the position number (Figure 6b). Subsequently, species‐specific SNP barcode patterns were generated (Figure 7).

## 4 DISCUSSION

Currently, the DNA barcoding is widely used in systematics and species identification in evolutionary, ecological, and conservation research (Austerlitz et al., 2009; DasGupta, Konwar, Mandoiu, & Shvartsman, 2005; Hebert et al., 2003; Kress, Garcia‐Robledo, Uriarte, & Erickson, 2015; Meier, Shiyang, Vaidya, & Ng, 2006). The original intention of “barcode” is designed to make a straightforward, reliable, and cheap tag to identify species. Such tag should be similar to tag the goods in the supermarket which can easily obtain the product information through barcodes (Hebert et al., 2003).

Based on given barcodes, our intention is to reduce the nonvariable and noninformative sequence information. For this purpose, we implement an algorithm to process known sequences and obtain the shortest SNP barcode for species identification.

In the present study, we are using COI sequences (~652 to 722 bps) to generate reliably much shorter genetic species tags that are based on the actual nucleotide differences (SNPs) of a given group of species. This way we do not have to consider the bulk of uninformative nucleotide sequences many species share in a pool of phylogenetically related taxa. The DTSB method applied here to a group of 17 pigeon species generates the shortest possible DNA barcode for species identification. Such SNP barcode sequences are easily obtained after sequence alignment; for example, the number of *M* bp for SNP barcode sequence may be identified from *N* species with COI sequences. Each SNP barcode sequence can reliably identify each species of a given taxon, and the SNP barcodes are generated from decision tree algorithm that searches for *P* nodes, where *P *= [*p*_min_, *p*_max_], *p*_min_ = *N*/4, *p*_max_ = *N* − 1, and P nodes can be repeatedly selected if needed. For example, we assume that 10 species are included for classifying by SNP barcoding. *P *= [3, 9] is calculated in the formula “*P *= [*p*_min_, *p*_max_].” It means that in the most parsimonious case it just needs 3 bp to identify 10 species. In the least informative case, this would need 9 bp.

A permutation of *M* bp of DNA sequence had a 4^*M*^ combination of nucleotides, where “4” represents the four available DNA nucleotides “A, T, C, and G.” It is a permutation with repetition problem described as $H_{N-1}^{M}=C_{N-1}^{N-1+M-1}$ when *P* is *p*_max_. In the example of the current study, the sequence data with 652~722 bp from 17 species (*N* = 17) are obtained through sequence alignment and they are trimmed into the same length for 652 bp. After removing the same nucleotides, nucleotides representing 185 bp (*M* = 185) are discovered as SNPs. These provide the possibility of combinational pattern permutation through the following: $H_{N-1}^{M}=C_{N-1}^{N-1+M-1}=C_{17-1}^{17-1+185-1}=C_{16}^{200}>16E+21.$ This calculation indicates that 17 species with 185 SNPs from the same aligned nucleotides can provide huge combinations, which is larger than the requirement for combination in the making rule of decision tree for correct classification in present study. Therefore, our proposed method can shorten the needs for SNP barcode encoding in species discrimination.

Moreover, the DTSB is functional for a monophylum, that is, a phylogenetically coherent group of species. Within 17 Columbidae species, the number of SNP for the phylogenetically distantly related species is commonly higher than for closer related species. Therefore, a DTSB had a high potential to classify species reliably, efficiently, at low cost, and with high‐throughput potential with a short species‐specific SNP barcode.

Although SNPs can easily be identified, some tasks for computation are still necessary for species identification. The decision tree algorithm (Pei et al., 2015; Quinlan, 1986) is characterized by its high‐sensitive property to variations in the training data (Weitschek, Fiscon, & Felici, 2014). Consequently, the decision tree algorithm may be suitable to classify the SNPs generated in the COI barcoding computation but warrants further validation.

The DTSB is limited to discriminate species within the aligned sequences of known species. It is not suitable to extend identified SNP barcodes for the purpose of unknown species identification. In the current study, DTSB is only performed using 17 known COI sequences of the pigeon family, Columbidae. However, this method allows for and is particularly useful to apply for much larger sample sizes. It may also apply to other COI sequences from the BOLD: The Barcode of Life Data System (Ratnasingham & Hebert, 2007) and other non‐COI barcoding systems. This holds for the kingdom plants where a universal DNA barcode is still undetermined. However, the CBOL Plant Working Group suggested the so‐called core barcode (rbcL+matK) from chloroplast DNA for land plants in general (CBOL Plant Working Group, 2009). Some more alternative barcodes (trnH‐psbA, ITS) and the RuBisCo gene of plants were used in plants before (Dong et al., 2014). The sequence of ITS (internal transcribed spacer) is commonly applied for fungi (Schoch et al., 2012; Seifert, 2009).

## 5 CONCLUSION

The full length of cytochrome oxidase 1 (COI) sequence is suitable for species identification and phylogenetic inference. However, the full‐length “tag” makes it unfriendly for species identification and authentication. To function as the supermarket tagging, we propose a DTSB method to generate the shortest SNP barcode of a given COI sequence to discriminate 17 Columbidae species through this tree decision algorithm. The computational loading for full length (~650 bp) has been reduced to a SNP barcode of about 11 bp. In the future, these species‐specific short SNP barcode may provide a reliable, faster species identification. The DTSB method is also flexible to apply to non‐COI sequences, like ITS, RuBisCo for tagging biota from other kingdoms than animals.

## ACKNOWLEDGMENTS

This work was supported by funds of the Ministry of Science and Technology, Taiwan (MOST 102‐2221‐E‐151‐024‐MY3 and MOST 104‐2320‐B‐037‐013‐MY3), the National Sun Yat‐sen University‐KMU Joint Research Project (#NSYSU‐KMU 106‐p001).

## COMPETING INTERESTS

The authors declare there are no competing interests.
