# Supplementary material for: Single nucleotide polymorphism barcoding of cytochrome c oxidase I sequences for discriminating 17 species of Columbidae by decision tree algorithm
Source: Ecol Evol. 2017 May 23;7(13):4717–25. doi: 10.1002/ece3.3045 (PMC5496562; doi:10.1002/ece3.3045)
Supplement: Supplementary file 1 [file ECE3-7-4717-s001.pdf]

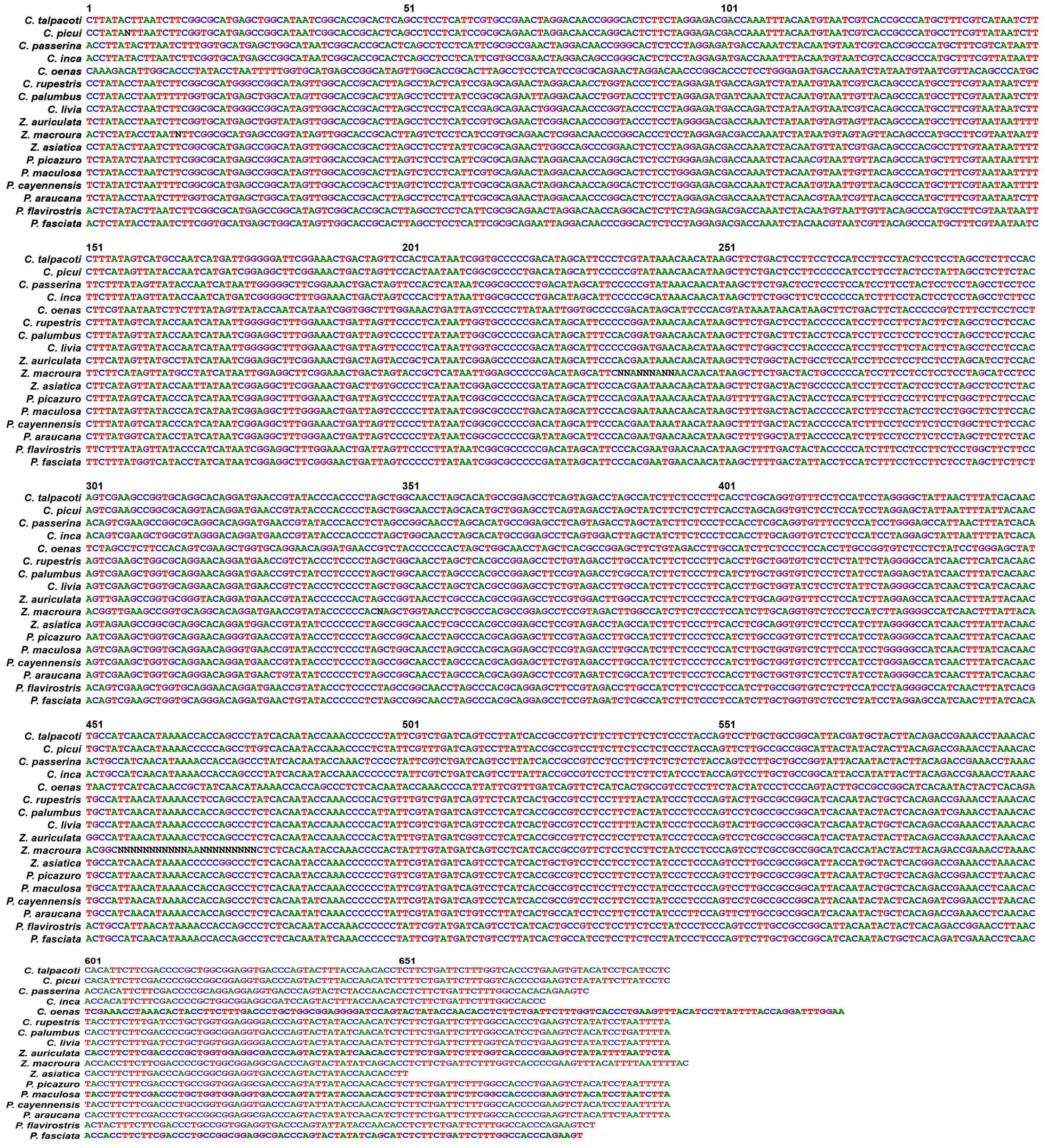
1
51
101
151
201
251
301
351
401
451
501
551
601
651
C. talpacoti
C. picui
C. passerina
C. inca
C. oenas
C. rupestris
C. palumbus
C. livia
Z. auriculata
Z. macroura
Z. asiatica
P. picazuro
P. maculosa
P. cayennensis
P. araucana
P. flavirostris
P. fasciata

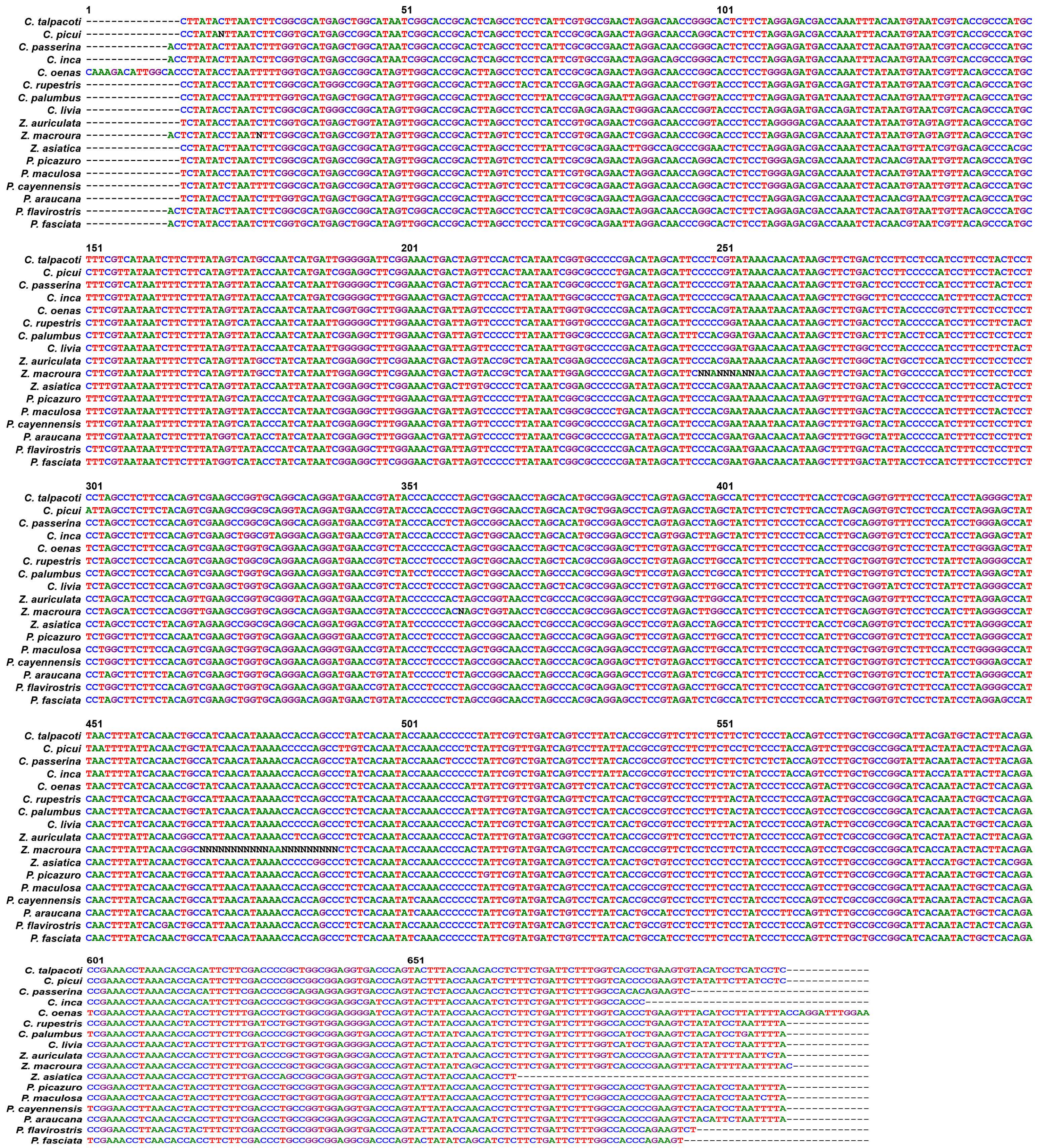

1
51
101
151
201
251
301
351
401
451
501
551
601
651
C. talpacoti
C. picui
C. passerina
C. inca
C. oenas
C. rupestris
C. palumbus
C. livia
Z. auriculata
Z. macroura
Z. asiatica
P. picazuro
P. maculosa
P. cayennensis
P. araucana
P. flavirostris
P. fasciata

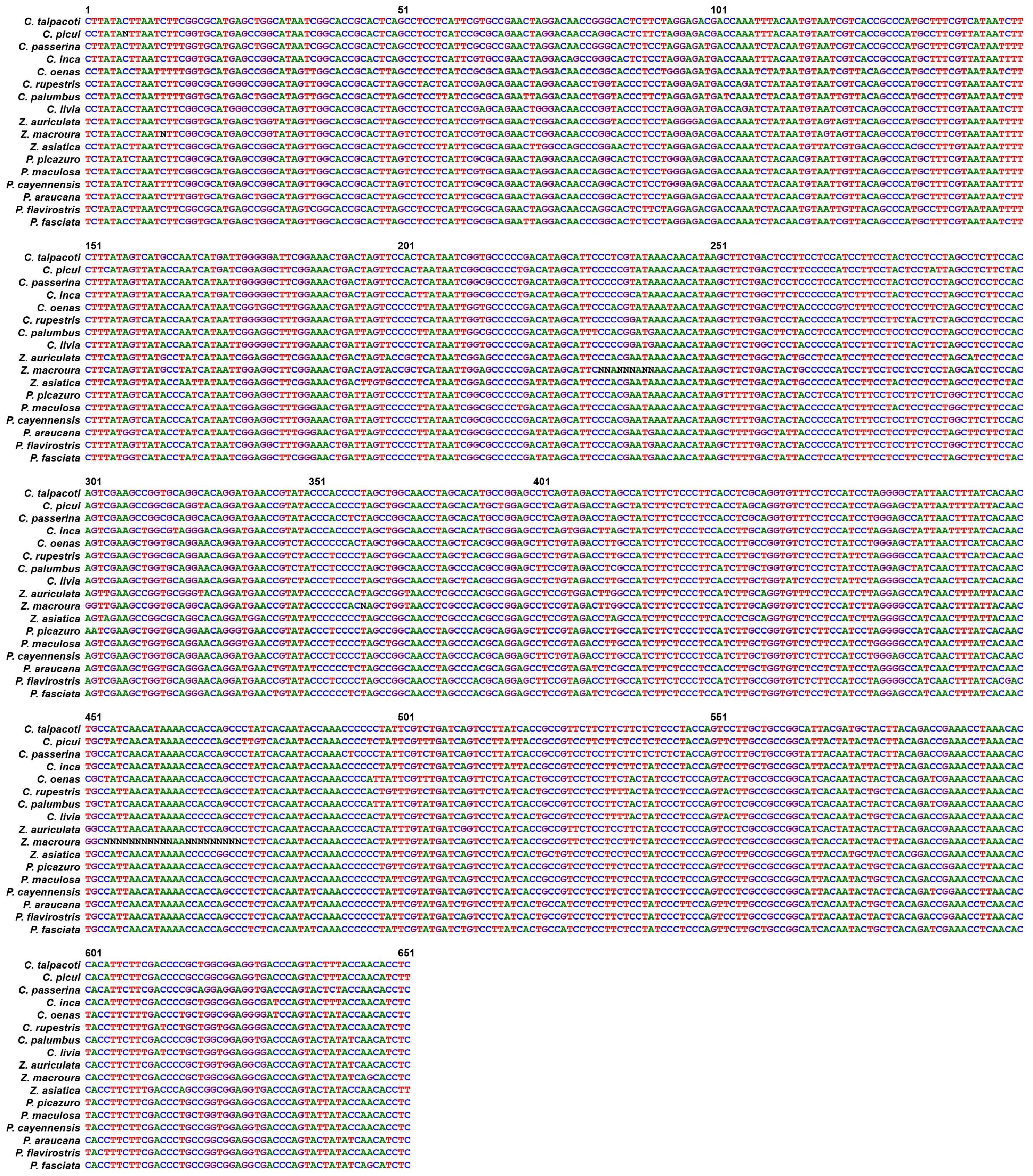
1
51
101
151
201
251
301
351
401
451
501
551
601
651
C. talpacoti
C. picui
C. passerina
C. inca
C. oenas
C. rupestris
C. palumbus
C. livia
Z. auriculata
Z. macroura
Z. asiatica
P. picazuro
P. maculosa
P. cayennensis
P. araucana
P. flavirostris
P. fasciata
